# A novel linked dataset to improve understanding about the health of people in Northern Ireland’s prisons

**DOI:** 10.23889/ijpds.v9i5.2696

**Published:** 2024-09-18

**Authors:** Janine Cooper, Dermot O’Reilly, Trish Kelly, Richard Kirk, Stephen McGarrigle, Ruth Gray, Michael Donnelly

**Affiliations:** 1 Queen's University Belfast; 2 Administrative Data Research Centre Northern Ireland; 3 South Eastern Health and Social Care Trust

## Objective and Approach

We describe a collaboration between the Queen’s University Belfast, Administrative Data Research Centre (ADRC) in Northern Ireland (NI) and the NI Healthcare in Prisons Service (HIPS) to develop a novel linked database to assess the health of people in prison in NI. Prison health data were extracted by the HIPS using electronic/manual modes for the period 2012-2021 and transferred to the Honest Broker Service, a trusted research environment, for linkage, de- identification and access to data. Data from prison health records were linked via a unique health identifier to a healthcare population spine, prescriptions data (primary care), hospital admissions data (mental health), income deprivation and mortality data.

## Results

A novel linked dataset has been developed including 16 variables from prison health records for N=14,898 individuals and N=34,213 custodial episodes (individuals may have ≥1 custodial episode during the study period). The entire NI population (healthcare population spine data) will be included as comparator data. Dataset cleaning has commenced for analyses. The study aims are to (i) examine the health/mental health of people in prison in NI, (ii) examine all-cause/drug-related mortality risk after release from prison and (iii) characterise individuals with the greatest mortality risk.

## Conclusions

We discuss the development of a novel linked dataset using prison health records and present results from our research questions about the health of people in prison.

## Implications

This work may direct future research and may be used to guide health services policy changes and improve outcomes after prison release.

